# Epistatic Interactions between Apolipoprotein E and Hemoglobin S Genes in Regulation of Malaria Parasitemia

**DOI:** 10.1371/journal.pone.0076924

**Published:** 2013-10-08

**Authors:** Virginie Rougeron, Caira M. Woods, Kathryn E. Tiedje, Florence Bodeau-Livinec, Florence Migot-Nabias, Philippe Deloron, Adrian J. F. Luty, Freya J. I. Fowkes, Karen P. Day

**Affiliations:** 1 Department of Microbiology, Division of Medical Parasitology, New York University School of Medicine, New York, New York, United States of America; 2 UMR216, Institut de Recherche pour le Développement, Paris, France; 3 PRES, Paris Sorbonne Cité, Université Paris Descartes, Paris, France; 4 Centre for Population Health, Macfarlane Burnet Institute of Medical Research and Public Health, Melbourne, Victoria, Australia; National University of Singapore, Singapore

## Abstract

Apolipoprotein E is a monomeric protein secreted by the liver and responsible for the transport of plasma cholesterol and triglycerides. The *APOE* gene encodes 3 isoforms Ɛ4, Ɛ3 and Ɛ2 with *APOE* Ɛ4 associated with higher plasma cholesterol levels and increased pathogenesis in several infectious diseases (HIV, HSV). Given that cholesterol is an important nutrient for malaria parasites, we examined whether *APOE* Ɛ4 was a risk factor for *Plasmodium* infection, in terms of prevalence or parasite density. A cross sectional survey was performed in 508 children aged 1 to 12 years in Gabon during the wet season. Children were screened for *Plasmodium* spp. infection, *APOE* and hemoglobin S (HbS) polymorphisms. Median parasite densities were significantly higher in *APOE* Ɛ4 children for *Plasmodium* spp. densities compared to non-*APOE* Ɛ4 children. When stratified for HbS polymorphisms, median *Plasmodium* spp. densities were significantly higher in HbAA children if they had an *APOE* Ɛ4 allele compared to those without an *APOE* Ɛ4 allele. When considering non-*APOE* Ɛ4 children, there was no quantitative reduction of *Plasmodium* spp. parasite densities for HbAS compared to HbAA phenotypes. No influence of *APOE* Ɛ4 on successful *Plasmodium* liver cell invasion was detected by multiplicity of infection. These results show that the *APOE* Ɛ4 allele is associated with higher median malaria parasite densities in children likely due to the importance of cholesterol availability to parasite growth and replication. Results suggest an epistatic interaction between *APOE* and *HbS* genes such that sickle cell trait only had an effect on parasite density in *APOE* Ɛ4 children. This suggests a linked pathway of regulation of parasite density involving expression of these genes. These findings have significance for understanding host determinants of regulation of malaria parasite density, the design of clinical trials as well as studies of co-infection with *Plasmodium* and other pathogens.

## Introduction

Children in malaria endemic areas experience multiple clinical episodes of malaria. They eventually develop acquired immunity that protects against clinical disease rather than infection. Clinical episodes occur against a background of chronic infections with multiple *Plasmodium* spp. and genotypes that can persist for months [1]. These chronic infections also represent a significant burden of disease as they contribute to anemia and can become symptomatic. Parasite density is the major determinant of whether an infection becomes symptomatic as evidenced by the fever threshold [2,3], a parasite density at which there is a >90% risk of having a malaria fever. The progression from asymptomatic infection to high parasite densities and associated clinical disease is determined by the interplay between a number of biological factors including acquired immunity, parasite virulence and host genetics.

A number of host polymorphisms have been shown to influence susceptibility to severe malarial disease [4] but there are surprisingly fewer examples of host genetics influencing *Plasmodium* blood stage infection levels *in vivo* [4,5]. Alpha-thalassemia, haptoglobin, complement receptor 1 and glucose 6- phosphate dehydrogenase polymorphisms moderate the outcome of severe disease but do not appear to influence parasite density [4-8]. In contrast, Duffy negative erythrocytes provide the classic example of generally total refractoriness to *P. vivax* in West Africa but not to *P. falciparum* infection [9-12]. Sickle cell hemoglobin (HbS) and hemoglobin C (HbC) appear to reduce *P. falciparum* infection levels as well as modify the clinical outcome of disease [4,13,14]. While the abovementioned polymorphisms have been intensively investigated in relation to malaria, little is known about whether polymorphisms in apolipoproteins, such as human apolipoprotein E, influence susceptibility to *Plasmodium* spp. infection.

Apolipoprotein E (ApoE for protein) is a monomeric protein secreted by the liver and responsible for the binding and removal of lipids and their remnants [15]. The apolipoprotein E gene (*APOE* for gene), located at chromosome 19q13.2, encodes 3 major alleles designated *APOE* Ɛ2, *APOE* Ɛ3 and *APOE* Ɛ4, defined by two single nucleotide polymorphisms (SNPs) located in exon 4 leading to different amino acids at positions 112 (Cys for Ɛ2 and Ɛ3, Arg for Ɛ4) and 158 (Arg for Ɛ3 and Ɛ4, Cys for Ɛ2) [16]. The three isoforms encoded by these alleles have been shown to have different functional and biochemical properties [17-20] and the efficiency of these proteins is heavily determined by genotype. The most common allele in the human population is *APOE* Ɛ3 [21]. However, *APOE* Ɛ4 is thought to be the ancestral allele that has been selected against over time [21,22]. *APOE* polymorphisms have been studied in relation to several human diseases, both non-communicable and infectious. The *APOE* Ɛ4 allele has been associated with an increased risk of Alzheimer’s disease, coronary heart disease and death after myocardial infarction [16,23-27] as well as several infectious diseases including human immunodeficiency virus HIV, hepatitis C and herpes simplex virus (HSV) [28,29].

The increased risk of disease in *APOE* Ɛ4 carriers is most likely due to differential blood cholesterol levels. It is well established in the cardiovascular literature that the *APOE* Ɛ4 allele is associated with elevated cholesterol compared to other alleles [15,26]. Specifically, the apoE4 proteins have been shown to be internalized and catabolized by the liver more rapidly than apoE2 and E3 isoforms, inducing a more rapid conversion of Very Low Density Lipoproteins (VLDL) to Low Density Lipoproteins (LDL) and resulting in increased cholesterol levels first in the liver and then in the plasma [19,26,30-33]. In addition, *APOE* Ɛ4 cells have also been shown to reduce fatty acid oxidation leading to accumulation of tissue and plasma lipids.

There are several potential interactions between *APOE* Ɛ4 and malaria. *Plasmodium* parasites are auxotrophic for host cholesterol [34-37], required for membrane synthesis and replication of blood and liver stages. This suggests that the increased bioavailability of cholesterol to *Plasmodium* spp. in *APOE* Ɛ4 carriers could give rise to higher parasite densities. In addition, *APOE* and *Plasmodium* sporozoites use the same receptors for cell entry leading to potential competition for binding to liver cells [38-41]. As apoE isoforms vary in strength of binding to liver cell receptors (apoE4 > E3 > E2) individuals with the *APOE* Ɛ4 allele could have less sporozoite infection in the liver [38-42] and a lower multiplicity of infection (MOI), a measure of *Plasmodium* spp. genotypes able to successfully infect and develop in the liver and succeed to patency in the blood.

To test these hypotheses, we investigated the association of *APOE* polymorphisms with *Plasmodium* spp. infection in children living in an area of seasonal malaria transmission in Southeastern Gabon. We compared *Plasmodium* spp. prevalence, density and *P. falciparum* MOI in children with different *APOE* alleles. In addition, sickle cell trait was also prevalent in this population [6] allowing us to investigate the potential interaction of this protective host erythrocyte polymorphism and *APOE* alleles in relation to *Plasmodium* spp. infection.

## Results

### Participant characteristics

A total of 508 asymptomatic children between the ages of 1-12 years were included in the current study investigating the association between *APOE* alleles/genotypes and *Plasmodium* spp. infection (see Table 1). 257 (50.6%) children were slide positive for any *Plasmodium* spp. with a median [IQR] *Plasmodium* spp. density of 800 [264-3060] parasites/µL. Most children were infected with *P. falciparum* (46.0%, n=234), and a few were infected with *P. malariae* (2.8%, n=14) and the prevalence of mixed *P. falciparum/ P. malariae* infection was only 1.8% (n=9). MOI was successfully genotyped for 206 of 234 *P. falciparum* positive samples and ranged from 1 to 4 (median = 2) with 112 (54.4%) infections being polyclonal (MOI > 1). Children aged 1-4 years had lower *Plasmodium* spp. prevalence (39.7%) compared to 5-9 (53.4%) and 10-12 (61.0%) year olds (*p* < 0.01). There was no significant association between age group as a categorical variable and parasite density (*p* ≥ 0.12), although previously we did report an association between age and parasite density as a continuous variable [6,7]. Older children had a median MOI of 2 compared to MOI of 1 for younger children (1-4 year olds) (*p* ≥ 0.24).

**Table 1:** Demographic, parasitologic and genetic characteristics of the study population.

**Category**	**Sub-Category**	**N (%)**
**Age Groups (N=508)**	1-4 years	136 (26.8)
	5-9 years	313 (61.6)
	10-12 years	59 (11.6)
**Sex (N=508)**	Female	235 (46.3)
	Male	273 (53.7)
***Plasmodium* spp. Prevalence (N=257)**	1-4 years	54 (39.7)
	5-9 years	167 (53.4)
	10-12 years	36 (61.0)
***Plasmodium* spp. Prevalence (N=257)**	*P. falciparum* positive	234 (46.0)
	*P. malariae* positive	14 (2.8)
	*P. falciparum/P. malariae* positive	9 (1.8)
***APOE* Alleles (N=508)**	Ɛ2	87 (17.1)
	Ɛ3	321 (63.2)
	Ɛ4	100 (19.7)
***APOE* Genotypes (N=508)**	Ɛ2/Ɛ2	27 (5.3)
	Ɛ2/Ɛ3	89 (17.5)
	Ɛ2/Ɛ4	31 (6.1)
	Ɛ3/Ɛ3	221 (43.5)
	Ɛ3/Ɛ4	111 (21.9)
	Ɛ4/Ɛ4	29 (5.7)
**Hb Phenotype** * **(N=461**)	HbAA	367 (79.6)
	HbAS	94 (20.4)

* 461 subjects were included for Hb analysis: exclusions included subjects who could not be phenotyped for Hb (n=44) and those with small sample numbers HbSS (n=3).

### Host genetic analysis

*APOE* alleles and HbS phenotype were successfully determined in 508 and 461 children respectively (Table 1). The frequencies of the *APOE* alleles in the study population were 17.1%, 63.2% and 19.7% for *APOE* Ɛ2, Ɛ3 and Ɛ4 respectively (Table 1). For *APOE* genotypes, *APOE* Ɛ3/Ɛ3 was the most prevalent genotype in this population (43.5%), followed by Ɛ3/Ɛ4 with 111 (21.9%) and Ɛ2/Ɛ3 with 89 (17.5%) (Table 1). *APOE* Ɛ2/Ɛ2, Ɛ2/Ɛ4 and Ɛ4/Ɛ4 genotypes were relatively rare (<7%, Table 1). Of the 461 children characterized for the HbS polymorphism, 20.4% of children had sickle cell trait (HbAS). The estimated *Hb* allelic frequencies were 89.8% for the A allele and 10.2% for the S allele.

### APOE and malariometric indices

The association between *APOE* alleles and malariometric indices was investigated. There were no significant associations between *APOE* alleles and the prevalence of either *Plasmodium* spp. (*p* > 0.24, Table 2), or *P. falciparum* (*p* > 0.28, Table 3) infection. There was also no association between *APOE* Ɛ3 or *APOE* Ɛ2 alleles with *Plasmodium* spp. (*p* > 0.25, Table 2) or *P. falciparum* (*p* > 0.41, Table 3) density. In contrast, as hypothesized, median parasite densities were significantly higher in *APOE* Ɛ4 children, compared to children who were non-*APOE* Ɛ4, for both total *Plasmodium* spp. (1280 vs. 640 parasite s/µL respectively, *p* = 0.04, Table 2) and *P. falciparum* (1373 vs. 631 parasite s/µL respectively, *p* = 0.02, Table 3). Interestingly, higher *P. malariae* parasite densities were also observed in children who were *APOE* Ɛ4 compared to non-*APOE* Ɛ4 (759 vs. 180 parasite s/µL respectively, *p* = 0.07). No significant associations were found with prevalence of MOI > 1 or median MOI with any of the *APOE* alleles (Table 4, *p* > 0.50) and HbAA or HbAS phenotypes (*p* > 0.82). When *APOE* alleles were further analyzed as genotypes, there was no significant difference in prevalence and density of either *Plasmodium* spp. or *P. falciparum* associated with any genotype although there was a trend of higher parasite densities in children having a genotype with one or more *APOE* Ɛ4 allele (data not shown).

**Table 2:** The asexual malaria parasite prevalence (n (%)) and median density (value/µL, Inter Quartile Range [IQR]) in relation to the *APOE* alleles for children positive for *Plasmodium* spp. (includes *P. falciparum*, *P. malariae, and mixed P. falciparum/P. malariae*) (N=257).

	***Plasmodium* spp. (N=257)**	
***APOE* Allele Groupings (N=508)**	**Prevalence (n/N (%))**	**Density (value/μL) median [IQR]**
***APOE* Ɛ4 (N=171)**	89 (52.1)	1280 [341-3680]
**non-*APOE* Ɛ4 (N=337)**	168 (49.9)	640 [204-2224]
***P-value***	0.64	0.04**
***APOE* Ɛ3 (N=421)**	208 (49.4)	753 [232-3134]
**non-*APOE* Ɛ3 (N=87)**	49 (56.3)	800 [320-2765]
***P-value***	0.24	0.66
***APOE* Ɛ2 (N=147)**	79 (53.7)	640 [167-2000]
**non-*APOE* Ɛ2 (N=361)**	178 (49.3)	800 [320-3151]
***P-value***	0.37	0.25

The chi-square test was used to compare proportions and the Mann-Whitney U test was used for variation across the two groups. Significant associations are noted “** ”.

**Table 3:** The asexual malaria parasite prevalence (n (%)) and median density (value/µL, Inter Quartile Range [IQR]) in relation to the *APOE* alleles for children only positive for *P. falciparum* (excludes *P. malariae, and mixed P. falciparum/P. malariae*) (N=234).

	***P. falciparum* (N=234)**	
***APOE* Allele Groupings (N=508)**	**Prevalence (n/N (%))**	**Density (value/μL) median [IQR]**
***APOE* Ɛ4 (N=171)**	79 (46.2)	1373 [362-4328]
**non-*APOE* Ɛ4 (N=337)**	155 (46.0)	631 [214-2227]
***P-value***	0.80	0.02**
***APOE* Ɛ3 (N=421)**	190 (45.1)	753 [238-3291]
**non-*APOE* Ɛ3 (N=87)**	44 (50.6)	800 [320-3000]
***P-value***	0.28	0.69
***APOE* Ɛ2 (N=147)**	72 (49.0)	640 [175-2942]
**non-*APOE* Ɛ2 (N=361)**	162 (44.9)	800 [320-3497]
***P-value***	0.37	0.41

The chi-square test was used to compare proportions and the Mann-Whitney U test was used for variation across the two groups. Significant associations are noted “** ”.

**Table 4:** The asexual malaria parasite MOI median (value, Inter Quartile Range [IQR]) and MOI>1 prevalence (n/N (%)) in relation to the *APOE* alleles for children only positive for *P. falciparum* (excludes *P. malariae, and mixed P. falciparum/P. malariae*) (N=206).

	***P. falciparum MOI* (N=206)**	
***APOE* Allele Groupings**	**MOI median [IQR]**	**Prevalence MOI>1 (n/N (%))**
***APOE* Ɛ4 (N=72)**	2 [1-2]	40/72 (55.6)
**non-*APOE* Ɛ4 (N=134)**	2 [1-2]	72/134 (53.7)
***P-value***		0.96
***APOE* Ɛ3 (N=166)**	2 [1-2]	94/166 (56.6)
**non-*APOE* Ɛ3 (N=40)**	2 [1-2]	18/40 (45.0)
***P-value***		0.50
***APOE* Ɛ2 (N=68)**	2 [1-2]	35/68 (51.5)
**non-*APOE* Ɛ2 (N=138)**	2 [1-2]	77/138 (55.8)
***P-value***		0.59

*The* chi-square test was used to compare proportions and the Mann-Whitney U test was used for variation across the two groups. Significant associations are noted “** ”.

### Interactions between the APOE Ɛ4 allele and HbAS

Overall, there was no association between HbAS phenotype and parasite prevalence (*p* > 0.46), but HbAA children had higher *Plasmodium* spp. densities compared to children who were HbAS (800 parasite s/µL vs. 480 parasite s/µL respectively, *p* = 0.05). To analyze whether HbAS could be an effect modifier on the observed associations between the *APOE* Ɛ4 allele and parasite densities, data were stratified for Hb phenotypes (Tables 5 and 6). Analysis of parasite density distributions revealed that children who were HbAA/*APOE* Ɛ4, had significantly higher *Plasmodium* spp. and *P. falciparum* densities compared to children who were HbAA/non-*APOE* Ɛ4 (Table 5, *p* = 0.01 and Table 6, *p* = 0.01 respectively). It was also observed that children who were HbAA/*APOE* Ɛ4 had significantly higher *Plasmodium* spp. and *P. falciparum* densities compared to children who were HbAS/*APOE* Ɛ4 (Table 5, *p* = 0.02 and Table 6, *p* = 0.05 respectively). Surprisingly, no significant difference in parasite density for both *Plasmodium* spp. and *P. falciparum* were observed for children who were non-*APOE* Ɛ4 and HbAS compared to those who were non-*APOE* Ɛ4 and HbAA (Table 5, *p* = 0.52 and Table 6, *p* = 0.50 respectively) i.e. there was no additive effect of HbAS on reducing parasite density in the absence of an *APOE* Ɛ4 allele.

**Table 5:** The distribution of parasite asexual median density (value/µL, Inter Quartile Range [IQR]) is based on the presence of the *APOE* Ɛ4 allele and subdivided by the modifier phenotypes: HbAA and HbAS, for children positive for *Plasmodium* spp. (includes *P. falciparum*, *P. malariae, and mixed P. falciparum/P. malariae*) (N=238).

		***Plasmodium* spp. (N=238)**		
		***APOE* Ɛ4 (N=83)**	**non-*APOE* Ɛ4 (N=155)**	***P-value***
**Hb Group Pooled**	**Density (value/μL) median [IQR]**	1280 [320-3680]	640 [217-2291]	0.05**
**HbAA (N=187)**	**n**	63	124	
	**Density (value/μL) median [IQR]**	1461 [501-4480]	640 [219-3070]	0.01**
**HbAS (N=51)**	**n**	20	31	
	**Density (value/μL) median [IQR]**	375 [116-2720]	640 [214-1977]	0.70
	***P-value***	0.02**	0.52	

The Mann-Whitney U test was used for evaluate variation across the two groups. Significant associations are noted “** ”.

**Table 6:** The distribution of parasite asexual median density (value/µL, Inter Quartile Range [IQR]) is based on the presence of the *APOE* Ɛ4 allele and subdivided by the modifier phenotypes: HbAA and HbAS, for children positive only for *P. falciparum* (excludes *P. malariae, and mixed P. falciparum/P. malariae*) (N=217).

		***P. falciparum* (N=217)**		
		***APOE* Ɛ4 (N=74)**	**non-*APOE* Ɛ4 (N=143)**	***P-value***
**Hb Group Pooled**	**Density (value/μL) median [IQR]**	1327 [352-4520]	631 [214-2227]	0.03**
**HbAA (N=169)**	**n**	56	113	
	**Density (value/μL) median [IQR]**	1462 [485-5600]	631 [223-3062]	0.01**
**HbAS (N=48)**	**n**	18	30	
	**Density (value/μL) median [IQR]**	434 [123-3120]	611 [201-1973]	0.98
	***P-value***	0.05**	0.50	

The Mann-Whitney U test was used for evaluate variation across the two groups. Significant associations are noted “** ”.

## Discussion

Data presented demonstrated a significant association between the *APOE* Ɛ4 allele and increased susceptibility to *Plasmodium* spp. infection in children exposed to intense seasonal malaria transmission. Indeed, substantially higher chronic *Plasmodium* spp. parasite densities (of both species) were observed in Gabonese children carrying the *APOE* Ɛ4 allele compared to those not having this allele. However, increased parasite density was not due to differential sporozoite competition for liver receptors because the number of infecting genomes (MOI) per child was not influenced by *APOE* Ɛ4 allele status. Thus, we propose that this increased level of parasite density results from greater cholesterol and fatty acid availability in hosts with an *APOE* Ɛ4 allele leading to increased membrane synthesis and parasite replication. This general nutrient availability mechanism of susceptibility to higher parasite densities would be a potential explanation of why the *APOE* Ɛ4 allele influences parasite density of all *Plasmodium* spp.

Another result of this study was the interaction between the *APOE* gene and *HbS* in relation to *Plasmodium* parasite density. Even if it is commonly observed that the children with the phenotype HbAS are characterized by lower parasite densities, this interaction would be best described as epistatic i.e. where the effects of one gene on the expression of a phenotype are modified by the presence of one or several other genes. Indeed, the *HbS* gene only had an effect of lowering parasite density in *APOE* Ɛ4 children as children who did not have an *APOE* Ɛ4 allele but were either *HbAA* or *HbAS* had similar low parasite densities. Hence, we conclude that the presence or absence of an *APOE* Ɛ4 allele had an overriding effect on parasite density. Importantly, we saw no additive effect of reducing parasite density in children who were *HbAS* and non-*APOE* Ɛ4. These data suggest the existence of complex epistatic interactions influencing a quantitative trait such as parasite density. Such interactions could also explain why *HbAS* is variably associated with lower parasite densities in field studies as the prevalence of the *APOE* Ɛ4 allele does vary among different study populations.

The observed epistatic interaction between genes involved in cholesterol and red blood cell (RBC) metabolism seemed intriguing and leads us to look for a linked pathway of regulation of parasite density influenced by both *APOE* and *HbS* to support the observation. Fairhurst and colleagues proposed that children with *HbAS* genotype have reduced infection levels because infected RBC of the *HbAS* genotype show lower expression of *P. falciparum* erythrocyte membrane protein 1 (PfEMP-1) the major variant surface antigen and the parasite ligand mediating cytoadherence, and reduced capacity to adhere [43]. A study by Frankland et al. provides a link between *APOE* and PfEMP-1 via cholesterol. They showed that depletion of cholesterol from RBC membrane inhibits the delivery or presentation of PfEMP-1 molecule to the RBC surface [44]. Similarly, Atorvastatin, a drug that lowers blood cholesterol decreases PfEMP-1 expression and cytoadherence to endothelial cells [45-47]. Consequently, in *APOE* Ɛ4 carriers more LDL and cholesterol is available to increase PfEMP-1 presentation to increase parasite survival and replication whereas those without *APOE* Ɛ4 will have lower PfEMP-1 presentation and lower parasite densities. Thus, we hypothesized that modulation of expression levels of PfEMP-1 provides a potential basis for an epistatic interaction between *APOE* and *HbS*. The overriding effect of *APOE* over *HbS* is most likely due to the role of cholesterol availability in expression of multiple phenotypes and not just PfEMP-1 expression.

This study was not designed to investigate the effect of *APOE* Ɛ4 polymorphisms on malaria morbidity outcomes. However, results revealed that *Plasmodium* spp. parasite densities were two to three times greater in children with the *APOE* Ɛ4 allele, which would increase the risk of both anemia and symptomatic malaria with parasite densities rising above the fever threshold [3]. Until today, only two field studies have investigated the association between *APOE* alleles with malaria outcomes [48,49]. Aucan et al. found no evidence for increased risk of severe malaria with any *APOE* allele in Gambian children, whereas Wozniak et al. showed that *APOE* Ɛ2/Ɛ2 was associated with early *P. falciparum* infection in infants in Ghana [48,49]. In addition to methodological heterogeneity, discrepancies between studies could be due to confounding from other host polymorphisms as observed in the current study. In our study, we demonstrated that specifically the *APOE* Ɛ4 allele was associated with higher median malaria parasite densities in West African children likely due to the importance of cholesterol availability to parasite growth and replication. To our knowledge, this is the first study involving a large enough sample size to investigate the association of *APOE* Ɛ4 alleles with the level of *Plasmodium* infection with consideration of confounding effects of sickle cell trait. Larger studies need to be completed in order to better explore the differential effect of *APOE* alleles and genotypes on susceptibility to clinical malarial disease stratifying for the confounding effect of *HbS* and potentially other host polymorphisms.

## Methods

### Study design and data collection

The study was performed in Bakoumba village, in Southeast Gabon near the Congo border. Malaria is highly endemic in this region with peaks of transmission at the end of the rainy seasons (September-December and March-June) [50]. A cross-sectional survey was conducted in May-June 2000 in 508 children 1-12 years of age. Details on the study population and data collection procedures have been published elsewhere [51]. Briefly, after obtaining informed consent from all parents, venous blood was collected in tubes containing EDTA for parasitological assessment for *Plasmodium* spp. by blood smears, HbS phenotyping and blood spots for genotyping [6,52]. For the present study, sufficient sample was available for *APOE* genotyping for 508 children. The study was reviewed and approved by the ethics committee of the International Center for Medical Research of Franceville, Gabon and New York University School of Medicine Ethical Review Board, United States of America.

### Parasitological measurement

Parasite densities were counted per 500 leukocytes on Giemsa-stained thick blood smears and were recorded as the number of parasites per microliter of blood, assuming the average leukocyte count was about 8000/µL [53]. Duplicate readings were made for a random 15% of smears to ensure quality control.

### Human genetic factors determination

The DNA was extracted from blood spots on filter paper using the QIAamp DNA Mini Kit (Qiagen, Valencia, CA). Sickle cell trait was detected by *Hb* electrophoresis [6,52]. *APOE* genotypes have been determined as published with modifications [54]. Two microliters of genomic DNA was amplified with 10µM of the published primers (upstream = 5’-TCC AAG GAG CTG CAG GCG GCG CA-3’, downstream= 5’-ACA GAA TTC GCC CCG GCC TGG TAC ACT GCC A-3’) [55] along with 2.5µL of Q solution, 12.5µL of 2X Master Mix from the Qiagen Multiplex PCR Kit (Qiagen, California, USA), zero point five microliters (10U/µL) of Cfo/enzyme (Promega) and water up to 25µL. Then, 1µL of its buffer were incubated with 3.5µL water, 0.1µL 100X BSA and 5µL of PCR product at 37°C for 1 hour. Products have been loaded in MetaPhore 4% agarose gel (Lonza Rockland, Inc., Maine, USA) in 1X TBE according to manufacturer’s instruction for electrophoresis.

### Multiple *P. falciparum* infections

Multiplicities of infection (MOI) represents a measure of *Plasmodium* spp. genotypes able to successfully infect and develop in the liver, and succeed to patency in the blood. In this study, MOI was determined for *P. falciparum* by MSP2 (Merozoite Surface Protein 2) nested PCR using published primers by Falk et al. with modifications (first round: MSP2-F1 = 5'-GAA GGT AAT TAA AAC ATT GTC-3' and MSP2-1R = 5'-ATG TTG CTG CTC CAC AG-3'; second round: M5 = 5'-GCA TTG CCA GAA CTT GAA-3', N5 = 5'-CTG AAG AGG TAC TGG TAG A-3' and STail = 5'-GTT TCT TCT TAT AAT ATG AGT ATA AGG AGA A-3') [56]. Duplicate readings have been made of reaction products visualised on 1.5% agarose gel stained with EnVISION^TM^ DNA Dye as Loading Buffer (Ambresco) to estimate the number of infections per sample.

### Statistical analysis

Associations between human genetic polymorphisms and malariametric indices (parasite prevalence, density, multiplicity of infection) were tested using non-parametric Mann-Whitney U test for continuous variables and by Chi-Square test for categorical variables. Statistical analyses were carried out using IBM SPSS Statistics Version 20 software.

For population genetic analysis, data were processed through Create V. 1.1. to convert the data for population genetics analyses [57]. We analyzed data with Fstat V. 2.9.3.2. software [58], updated from [59], which computes, estimates and tests the significance of various population genetic parameters. In this study, allele and genotype frequencies were estimated for *APOE* genotypes and *Hb* genotypes inferred from phenotypes.

For the analyses investigating the association between *APOE* genotypes/alleles with parasitological factors (density/prevalence), 508 asymptomatic children were included. For the analyses investigating the interaction between *APOE* alleles and Hb phenotypes together with parasitological factors (density/prevalence), 461 subjects from the cohort of 508 were included. Exclusions included subjects who could not be phenotyped for Hb (n = 44) as a result of limited blood sample collection and those with small sample numbers such as HbSS (n = 3). There were no statistical differences between the 461 children included for the *APOE* and Hb analyses and those who were not included in regards to the other variables (*p* > 0.05).

## Conclusions

In summary, we have identified *APOE* Ɛ4 as a significant host genetic modifier of malaria parasite density in West African children. The most likely explanation for this association is cholesterol availability for parasite replication in the liver and blood. In addition, we observed an epistatic interaction between *APOE* and *HbS* genes in relation to regulation of malaria parasite density indicating a potential linked pathway of regulation of parasite density, possibly by modulating expression of the *P. falciparum major* variant surface antigen. These findings have significance for understanding host determinants of regulation of malaria parasite density, the design of clinical trials as well as studies of co-infection with malaria and other pathogens. Given a fitness cost to higher parasite densities, our data are consistent with the proposal that malaria may have selected against the *APOE* Ɛ4 allele [60].

## References

[1] Bruce MC, Galinski MR, Barnwell JW, Donnelly CA, Walmsley M et al. (2000) Genetic diversity and dynamics of *Plasmodium* *falciparum* and *P.* *vivax* populations in multiply infected children with asymptomatic malaria infections in Papua New Guinea. Parasitology 121(Pt 3): 257-272. doi:10.1017/S0031182099006356. PubMed: 11085246. 11085246

[2] Rogier C (2000) Natural history of *Plasmodium* *falciparum* malaria and determining factors of the acquisition of antimalaria immunity in two endemic areas, Dielmo and Ndiop (Senegal). Bull Mem Acad R Med Belg 155: 218-226. PubMed: 11304957. 11304957

[3] Rogier C, Commenges D, Trape JF (1996) Evidence for an age-dependent pyrogenic threshold of *Plasmodium* *falciparum* parasitemia in highly endemic populations. Am J Trop Med Hyg 54: 613-619. PubMed: 8686780. 8686780 10.4269/ajtmh.1996.54.613

[4] Kwiatkowski DP (2005) How malaria has affected the human genome and what human genetics can teach us about malaria. Am J Hum Genet 77: 171-192. doi:10.1086/432519. PubMed: 16001361. 16001361 PMC1224522

[5] Taylor SM, Parobek CM, Fairhurst RM (2012) Haemoglobinopathies and the clinical epidemiology of malaria: a systematic review and meta-analysis. Lancet Infect Dis 12: 457-468. doi:10.1016/S1473-3099(12)70055-5. PubMed: 22445352. 22445352 PMC3404513

[6] Fowkes FJ, Imrie H, Migot-Nabias F, Michon P, Justice A et al. (2006) Association of haptoglobin levels with age, parasite density, and haptoglobin genotype in a malaria-endemic area of Gabon. Am J Trop Med Hyg 74: 26-30. PubMed: 16407342. 16407342

[7] Fowkes FJ, Michon P, Pilling L, Ripley RM, Tavul L et al. (2008) Host erythrocyte polymorphisms and exposure to *Plasmodium* *falciparum* in Papua New Guinea. Malar J 7: 1. doi:10.1186/1475-2875-7-1. PubMed: 18173836. 18173836 PMC2235880

[8] Imrie H, Fowkes FJ, Michon P, Tavul L, Hume JC et al. (2006) Haptoglobin levels are associated with haptoglobin genotype and alpha+ -Thalassemia in a malaria-endemic area. Am J Trop Med Hyg 74: 965-971. PubMed: 16760505. 16760505

[9] Horuk R, Chitnis CE, Darbonne WC, Colby TJ, Rybicki A et al. (1993) A receptor for the malarial parasite *Plasmodium* *vivax*: the erythrocyte chemokine receptor. Science 261: 1182-1184. doi:10.1126/science.7689250. PubMed: 7689250. 7689250

[10] Kasehagen LJ, Mueller I, Kiniboro B, Bockarie MJ, Reeder JC et al. (2007) Reduced *Plasmodium* *vivax* erythrocyte infection in PNG Duffy-negative heterozygotes. PLOS ONE 2: e336. doi:10.1371/journal.pone.0000336. PubMed: 17389925. 17389925 PMC1829178

[11] Ménard D, Barnadas C, Bouchier C, Henry-Halldin C, Gray LR et al. (2010) *Plasmodium* *vivax* clinical malaria is commonly observed in Duffy-negative Malagasy people. Proc Natl Acad Sci U S A 107: 5967-5971. doi:10.1073/pnas.0912496107. PubMed: 20231434. 20231434 PMC2851935

[12] Zimmerman PA, Woolley I, Masinde GL, Miller SM, McNamara DT et al. (1999) Emergence of FY*A(null) in a *Plasmodium* *vivax*-endemic region of Papua New Guinea. Proc Natl Acad Sci U S A 96: 13973-13977. doi:10.1073/pnas.96.24.13973. PubMed: 10570183. 10570183 PMC24175

[13] Mackinnon MJ, Mwangi TW, Snow RW, Marsh K, Williams TN (2005) Heritability of malaria in Africa. PLOS Med 2: e340. doi:10.1371/journal.pmed.0020340. PubMed: 16259530. 16259530 PMC1277928

[14] Solovieff N, Milton JN, Hartley SW, Sherva R, Sebastiani P et al. (2010) Fetal hemoglobin in sickle cell anemia: genome-wide association studies suggest a regulatory region in the 5' olfactory receptor gene cluster. Blood 115: 1815-1822. doi:10.1182/blood-2009-08-239517. PubMed: 20018918. 20018918 PMC2832816

[15] Ribalta J, Vallvé JC, Girona J, Masana L (2003) Apolipoprotein and apolipoprotein receptor genes, blood lipids and disease. Curr Opin Clin Nutr Metab Care 6: 177-187. doi:10.1097/00075197-200303000-00006. PubMed: 12589187. 12589187

[16] Siest G, Pillot T, Régis-Bailly A, Leininger-Muller B, Steinmetz J et al. (1995) Apolipoprotein E: an important gene and protein to follow in laboratory medicine. Clin Chem 41: 1068-1086. PubMed: 7628082. 7628082

[17] Boerwinkle E, Utermann G (1988) Simultaneous effects of the apolipoprotein E polymorphism on apolipoprotein E, apolipoprotein B, and cholesterol metabolism. Am J Hum Genet 42: 104-112. PubMed: 3337104. 3337104 PMC1715322

[18] Gregg RE, Brewer HB Jr. (1988) The role of apolipoprotein E and lipoprotein receptors in modulating the in vivo metabolism of apolipoprotein B-containing lipoproteins in humans. Clin Chem 34: B28-B32. PubMed: 2841050. 2841050

[19] Mamotte CD, Sturm M, Foo JI, van Bockxmeer FM, Taylor RR (1999) Comparison of the LDL-receptor binding of VLDL and LDL from apoE4 and apoE3 homozygotes. Am J Physiol 276: E553-E557. PubMed: 10070023. 10070023 10.1152/ajpendo.1999.276.3.E553

[20] Weisgraber KH (1994) Apolipoprotein E: structure-function relationships. Adv Protein Chem 45: 249-302. doi:10.1016/S0065-3233(08)60642-7. PubMed: 8154371. 8154371

[21] Corbo RM, Scacchi R (1999) Apolipoprotein E (APOE) allele distribution in the world. Is APOE*4 a 'thrifty' allele? Ann Hum Genet 63: 301-310. doi:10.1046/j.1469-1809.1999.6340301.x. PubMed: 10738542. 10738542

[22] Hanlon CS, Rubinsztein DC (1995) Arginine residues at codons 112 and 158 in the apolipoprotein E gene correspond to the ancestral state in humans. Atherosclerosis 112: 85-90. doi:10.1016/0021-9150(94)05402-5. PubMed: 7772071. 7772071

[23] Corder EH, Saunders AM, Strittmatter WJ, Schmechel DE, Gaskell PC et al. (1993) Gene dose of apolipoprotein E type 4 allele and the risk of Alzheimer’s disease in late onset families. Science 261: 921-923. doi:10.1126/science.8346443. PubMed: 8346443. 8346443

[24] Gerdes LU, Gerdes C, Kervinen K, Savolainen M, Klausen IC et al. (2000) The apolipoprotein epsilon4 allele determines prognosis and the effect on prognosis of simvastatin in survivors of myocardial infarction : a substudy of the Scandinavian simvastatin survival study. Circulation 101: 1366-1371. doi:10.1161/01.CIR.101.12.1366. PubMed: 10736278. 10736278

[25] Katzman R (1994) Apolipoprotein E and Alzheimer’s disease. Curr Opin Neurobiol 4: 703-707. doi:10.1016/0959-4388(94)90013-2. PubMed: 7849527. 7849527

[26] Mahley RW, Rall SC Jr. (2000) Apolipoprotein E: far more than a lipid transport protein. Annu Rev Genomics Hum Genet 1: 507-537. doi:10.1146/annurev.genom.1.1.507. PubMed: 11701639. 11701639

[27] Corder EH, Blennow K, Prince JA (2008) Genetic susceptibility sets for Alzheimer’s disease identified from diverse candidate loci. Rejuvenation Res 11: 667-679. doi:10.1089/rej.2008.0742. PubMed: 18593285. 18593285

[28] Corder EH, Paganelli R, Giunta S, Franceschi C (2008) Differential course of HIV-1 infection and APOE polymorphism. Proc Natl Acad Sci U S A 105: E87. doi:10.1073/pnas.0808164105. PubMed: 19004794. 19004794 PMC2584746

[29] Kuhlmann I, Minihane AM, Huebbe P, Nebel A, Rimbach G (2010) Apolipoprotein E genotype and hepatitis C, HIV and herpes simplex disease risk: a literature review. Lipids Health Dis 9: 8. doi:10.1186/1476-511X-9-8. PubMed: 20109174. 20109174 PMC2830997

[30] Gregg RE, Brewer HB Jr. (1986) In vivo metabolism of apolipoprotein E in humans. Methods Enzymol 129: 482-497. doi:10.1016/0076-6879(86)29087-4. PubMed: 3523156. 3523156

[31] Lucotte G, Loirat F, Hazout S (1997) Pattern of gradient of apolipoprotein E allele *4 frequencies in western Europe. Hum Biol 69: 253-262. PubMed: 9057348. 9057348

[32] Utermann G, Kindermann I, Kaffarnik H, Steinmetz A (1984) Apolipoprotein E phenotypes and hyperlipidemia. Hum Genet 65: 232-236. doi:10.1007/BF00286508. PubMed: 6698547. 6698547

[33] Weintraub MS, Eisenberg S, Breslow JL (1987) Dietary fat clearance in normal subjects is regulated by genetic variation in apolipoprotein E. J Clin Invest 80: 1571-1577. doi:10.1172/JCI113243. PubMed: 3479440. 3479440 PMC442425

[34] Bansal D, Bhatti HS, Sehgal R (2005) Role of cholesterol in parasitic infections. Lipids Health Dis 4: 10. doi:10.1186/1476-511X-4-10. PubMed: 15882457. 15882457 PMC1142336

[35] Jayabalasingham B, Bano N, Coppens I (2010) Metamorphosis of the malaria parasite in the liver is associated with organelle clearance. Cell Res 20: 1043-1059. doi:10.1038/cr.2010.88. PubMed: 20567259. 20567259 PMC4137911

[36] Jayabalasingham B, Menard R, Fidock DA (2010) Recent insights into fatty acid acquisition and metabolism in malarial parasites. F1000. Biol Reprod 2. 10.3410/B2-24 PMC2948358 20948809

[37] Labaied M, Jayabalasingham B, Bano N, Cha SJ, Sandoval J et al. (2011) *Plasmodium* salvages cholesterol internalized by LDL and synthesized de novo in the liver. Cell Microbiol 13: 569-586. doi:10.1111/j.1462-5822.2010.01555.x. PubMed: 21105984. 21105984

[38] Rathore D, Kumar S, Lanar DE, McCutchan TF (2001) Disruption of disulfide linkages of the *Plasmodium* *falciparum* circumsporozoite protein: effects on cytotoxic and antibody responses in mice. Mol Biochem Parasitol 118: 75-82. doi:10.1016/S0166-6851(01)00369-3. PubMed: 11704275. 11704275

[39] Rathore D, Sacci JB, de la Vega P, McCutchan TF (2002) Binding and invasion of liver cells by *Plasmodium* *falciparum* sporozoites. Essential involvement of the amino terminus of circumsporozoite protein. J Biol Chem 277: 7092-7098. doi:10.1074/jbc.M106862200. PubMed: 11751898. 11751898

[40] Shakibaei M, Frevert U (1996) Dual interaction of the malaria circumsporozoite protein with the low density lipoprotein receptor-related protein (LRP) and heparan sulfate proteoglycans. J Exp Med 184: 1699-1711. doi:10.1084/jem.184.5.1699. PubMed: 8920859. 8920859 PMC2192891

[41] Sinnis P, Willnow TE, Briones MR, Herz J, Nussenzweig V (1996) Remnant lipoproteins inhibit malaria sporozoite invasion of hepatocytes. J Exp Med 184: 945-954. doi:10.1084/jem.184.3.945. PubMed: 9064354. 9064354 PMC2192800

[42] Vignali M, McKinlay A, LaCount DJ, Chettier R, Bell R et al. (2008) Interaction of an atypical *Plasmodium* *falciparum* ETRAMP with human apolipoproteins. Malar J 7: 211. doi:10.1186/1475-2875-7-211. PubMed: 18937849. 18937849 PMC2577112

[43] Fairhurst RM, Wellems TE (2006) Modulation of malaria virulence by determinants of *Plasmodium* *falciparum* erythrocyte membrane protein-1 display. Curr Opin Hematol 13: 124-130. doi:10.1097/01.moh.0000219655.73162.42. PubMed: 16567953. 16567953

[44] Frankland S, Adisa A, Horrocks P, Taraschi TF, Schneider T et al. (2006) Delivery of the malaria virulence protein PfEMP1 to the erythrocyte surface requires cholesterol-rich domains. Eukaryot Cell 5: 849-860. doi:10.1128/EC.5.5.849-860.2006. PubMed: 16682462. 16682462 PMC1459682

[45] Parquet V, Briolant S, Torrentino-Madamet M, Henry M, Almeras L et al. (2009) Atorvastatin is a promising partner for antimalarial drugs in treatment of *Plasmodium* *falciparum* malaria. Antimicrob Agents Chemother 53: 2248-2252. doi:10.1128/AAC.01462-08. PubMed: 19307369. 19307369 PMC2687225

[46] Parquet V, Henry M, Wurtz N, Dormoi J, Briolant S et al. (2010) Atorvastatin as a potential anti-malarial drug: in vitro synergy in combinational therapy with quinine against *Plasmodium* *falciparum* . Malar J 9: 139. doi:10.1186/1475-2875-9-139. PubMed: 20497586. 20497586 PMC2882376

[47] Taoufiq Z, Pino P, N’Dilimabaka N, Arrouss I, Assi S et al. (2011) Atorvastatin prevents Plasmodium falciparum cytoadherence and endothelial damage. Malar J 10: 52. doi:10.1186/1475-2875-10-52. PubMed: 21356073. 21356073 PMC3056843

[48] Aucan C, Walley AJ, Hill AV (2004) Common apolipoprotein E polymorphisms and risk of clinical malaria in the Gambia. J Med Genet 41: 21-24. doi:10.1136/jmg.2003.012104. PubMed: 14729824. 14729824 PMC1757275

[49] Wozniak MA, Faragher EB, Todd JA, Koram KA, Riley EM et al. (2003) Does apolipoprotein E polymorphism influence susceptibility to malaria? J Med Genet 40: 348-351. doi:10.1136/jmg.40.5.348. PubMed: 12746397. 12746397 PMC1735474

[50] Elissa N, Migot-Nabias F, Luty A, Renaut A, Touré F et al. (2003) Relationship between entomological inoculation rate, *Plasmodium* *falciparum* prevalence rate, and incidence of malaria attack in rural Gabon. Acta Trop 85: 355-361. doi:10.1016/S0001-706X(02)00266-8. PubMed: 12659973. 12659973

[51] Ntoumi F, Ekala MT, Makuwa M, Lekoulou F, Mercereau-Puijalon O et al. (2002) Sickle cell trait carriage: imbalanced distribution of IgG subclass antibodies reactive to *Plasmodium* *falciparum* family-specific MSP2 peptides in serum samples from Gabonese children. Immunol Lett 84: 9-16. doi:10.1016/S0165-2478(02)00131-1. PubMed: 12161278. 12161278

[52] Migot-Nabias F, Mombo LE, Luty AJ, Dubois B, Nabias R et al. (2000) Human genetic factors related to susceptibility to mild malaria in Gabon. Genes Immun 1: 435-441. doi:10.1038/sj.gene.6363703. PubMed: 11196674. 11196674

[53] Cox MJ, Kum DE, Tavul L, Narara A, Raiko A et al. (1994) Dynamics of malaria parasitaemia associated with febrile illness in children from a rural area of Madang, Papua New Guinea. Trans R Soc Trop Med Hyg 88: 191-197. doi:10.1016/0035-9203(94)90292-5. PubMed: 8036670. 8036670

[54] Crook R, Hardy J, Duff K (1994) Single-day apolipoprotein E genotyping. J Neurosci Methods 53: 125-127. doi:10.1016/0165-0270(94)90168-6. PubMed: 7823614. 7823614

[55] Wenham PR, Price WH, Blandell G (1991) Apolipoprotein E genotyping by one-stage PCR. Lancet 337: 1158-1159. doi:10.1016/0140-6736(91)92823-K. PubMed: 1674030. 1674030

[56] Falk N, Maire N, Sama W, Owusu-Agyei S, Smith T et al. (2006) Comparison of PCR-RFLP and Genescan-based genotyping for analyzing infection dynamics of *Plasmodium* *falciparum* . Am J Trop Med Hyg 74: 944-950. PubMed: 16760501. 16760501

[57] Coombs JA, Letcher BH, Nislow KH (2008) create: a software to create input files from diploid genotypic data for 52 genetic software programs. Mol Ecol Resour 8: 578-580. doi:10.1111/j.1471-8286.2007.02036.x. PubMed: 21585837. 21585837

[58] Goudet J (2002) STAT, aprogram to estimate and test gene diversities and fixation indices (version 2.9.3).

[59] Goudet J (1995) FSTAT (Version 1.2): A computer program to calculate F-statistics. J Hered 86: 485-486.

[60] Eisenberg DT, Kuzawa CW, Hayes MG (2010) Worldwide allele frequencies of the human apolipoprotein E gene: climate, local adaptations, and evolutionary history. Am J Phys Anthropol 143: 100-111. PubMed: 20734437. 20734437 10.1002/ajpa.21298

